# Supplementary material for: The impact of changing cigarette smoking habits and smoke-free legislation on orofacial cleft incidence in the United Kingdom: Evidence from two time-series studies
Source: PLoS One. 2021 Nov 24;16(11):e0259820. doi: 10.1371/journal.pone.0259820 (PMC8612573; doi:10.1371/journal.pone.0259820)
Supplement: S1 Appendix — (DOCX) [file pone.0259820.s001.docx]

**S1 Appendix: Projections of orofacial cleft incidence**

Calculation of predicted numbers of annual orofacial cleft births in the UK, projected from actual UK orofacial cleft births in year 2000. Population-level projections calculated using the odds ratio to describe the association between active maternal smoking and orofacial cleft from individual-level studies (Fell M, Dack K, Chummun S, et al*.* Maternal cigarette smoking and cleft lip and palate: A systematic review and meta-analysis. *Cleft Palate Craniofac J*. 2021. Epub ahead of print)

**Terms used :**

- PAF = Proportional Attributable Fraction – in this context we are referring to the impact of smoking on orofacial cleft incidence
- Y2K = the year 2000, which is the baseline year
- PAFY2K = PAF in the baseline year of 2000
- PAFIncY2K = the cleft incidence attributable to smoking according to the PAF
- PredInc = predicted cleft incidence (per 10,000 live births) based on the PAF
- PredCount = predicted number (count) of clefts born each year, based on the PredInc

**Calculation:**

1. Effect estimates extracted from recently performed meta-analysis (OR with 95% CI)
2. PAF = prevalence of exposure *(1-1/OR)

PAF can be calculated for each year 2000-2018, with changes based on changing prevalence of exposure

1. PAFY2K = (30.56/100)*(1-1/OR)
2. PAFIncY2K = PAFY2K * cleft incidence in year 2000
3. PredInc = cleft incidence in 2000 – (PAFInc2K– PAFInc2K * (PAF/PAFY2K))
4. PredCount = PredInc/10,000 * number of annual live births

**Worked example:** PAF predicted OFC counts in England, Wales and Northern Ireland.

1. Effect estimate for OFC from Fell et al meta-analysis = 1.42
2. PAF can be calculated for each year (see table below)
3. PAFY2K = (30.56/100)*(1-1/1.42)

PAFY2K = 0.09

1. PAFIncY2K = 0.09*14.86

PAFIncY2K = 1.34

1. PredInc can be calculated for each year (see table A and B below)
2. PredCount can be calculated for each year (see table A and B below)

Calculations can be extrapolated for data in Scotland using the dataset available in S2 Appendix.

**Table A:** Projected annual incidence and count of Orofacial Cleft (OFC) births in England, Wales and Northern Ireland (EWNI) based on Proportional Attributable Fraction (PAF) projections from Exposure Proxy 1 using 2000 as the base year. Exposure Proxy 1 is the proportion of active smokers in females over 16 years of age in the UK reported by calendar year.

| **Year** | **Live BIRTHS EWNI** | **Actual OFC Count** | **Actual OFC incidence per 10,000 live births** | **Exposure Proxy 1**  **Active Smokes( %)** | **PAF**  **(When OR = 1.42)** | **Projected OFC incidence (PredInc)** | **Projected OFC count (PredCount)** |
| --- | --- | --- | --- | --- | --- | --- | --- |
| 2000 | 625953 | 930 | 14.86 | 30.6 | 0.09 | 14.86 | 930 |
| 2001 | 616596 | 951 | 15.42 | 30.6 | 0.09 | 14.86 | 916 |
| 2002 | 617507 | 884 | 14.32 | 31.4 | 0.09 | 14.89 | 920 |
| 2003 | 643117 | 917 | 14.26 | 29.9 | 0.09 | 14.83 | 954 |
| 2004 | 662039 | 970 | 14.65 | 28.0 | 0.08 | 14.74 | 976 |
| 2005 | 668163 | 1067 | 15.97 | 27.6 | 0.08 | 14.73 | 984 |
| 2006 | 692873 | 1094 | 15.79 | 25.2 | 0.07 | 14.62 | 1013 |
| 2007 | 714464 | 1147 | 16.05 | 23.8 | 0.07 | 14.56 | 1040 |
| 2008 | 734342 | 1177 | 16.03 | 25.8 | 0.08 | 14.65 | 1076 |
| 2009 | 731158 | 1044 | 14.28 | 24.4 | 0.07 | 14.58 | 1066 |
| 2010 | 748480 | 1062 | 14.19 | 23.9 | 0.07 | 14.57 | 1090 |
| 2011 | 749186 | 1107 | 14.78 | 22.8 | 0.07 | 14.52 | 1088 |
| 2012 | 754943 | 1168 | 15.47 | 22.2 | 0.07 | 14.49 | 1094 |
| 2013 | 722789 | 1167 | 16.15 | 20.1 | 0.06 | 14.40 | 1041 |
| 2014 | 719627 | 1073 | 14.91 | 20.8 | 0.06 | 14.43 | 1038 |
| 2015 | 722067 | 1119 | 15.50 | 21.4 | 0.06 | 14.45 | 1044 |
| 2016 | 720347 | 1073 | 14.90 | 17.5 | 0.05 | 14.28 | 1029 |
| 2017 | 702181 | 1081 | 15.39 | 18.5 | 0.05 | 14.33 | 1006 |
| 2018 | 679905 | 1018 | 14.97 | 19.2 | 0.06 | 14.36 | 976 |

**Table B:** Projected annual incidence and count of Orofacial Cleft (OFC) births in England, Wales and Northern Ireland (EWNI) based on Proportional Attributable Fraction (PAF) projections from Exposure Proxy 2 using 2000 as the base year. Exposure Proxy 2 is the proportion of active smokers in pregnant women in Scotland attending antenatal booking appointment reported by financial year.

| **Year** | **Live BIRTHS EWNI** | **Actual OFC Count** | **Actual OFC incidence per 10,000 live births** | **Exposure Proxy 2**  **Active Smokes( %)** | **PAF**  **(When OR = 1.42)** | **Projected OFC incidence (PredInc)** | **Projected OFC count (PredCount)** |
| --- | --- | --- | --- | --- | --- | --- | --- |
| 2000 | 625953 | 930 | 14.86 | 28.9 | 0.09 | 14.86 | 930 |
| 2001 | 616596 | 951 | 15.42 | 29.3 | 0.09 | 14.87 | 917 |
| 2002 | 617507 | 884 | 14.32 | 28.5 | 0.09 | 14.84 | 916 |
| 2003 | 643117 | 917 | 14.26 | 27.5 | 0.08 | 14.80 | 952 |
| 2004 | 662039 | 970 | 14.65 | 25.9 | 0.08 | 14.73 | 975 |
| 2005 | 668163 | 1067 | 15.97 | 24.3 | 0.07 | 14.66 | 979 |
| 2006 | 692873 | 1094 | 15.79 | 23.9 | 0.07 | 14.64 | 1014 |
| 2007 | 714464 | 1147 | 16.05 | 23.7 | 0.07 | 14.63 | 1045 |
| 2008 | 734342 | 1177 | 16.03 | 22.3 | 0.07 | 14.57 | 1070 |
| 2009 | 731158 | 1044 | 14.28 | 21.1 | 0.06 | 14.51 | 1061 |
| 2010 | 748480 | 1062 | 14.19 | 21.0 | 0.06 | 14.51 | 1086 |
| 2011 | 749186 | 1107 | 14.78 | 20.2 | 0.06 | 14.48 | 1084 |
| 2012 | 754943 | 1168 | 15.47 | 20.4 | 0.06 | 14.48 | 1093 |
| 2013 | 722789 | 1167 | 16.15 | 19.4 | 0.06 | 14.44 | 1044 |
| 2014 | 719627 | 1073 | 14.91 | 18.3 | 0.05 | 14.39 | 1034 |
| 2015 | 722067 | 1119 | 15.50 | 17.8 | 0.05 | 14.37 | 1038 |
| 2016 | 720347 | 1073 | 14.90 | 15.9 | 0.05 | 14.29 | 1029 |
| 2017 | 702181 | 1081 | 15.39 | 15.0 | 0.04 | 14.25 | 1000 |
| 2018 | 679905 | 1018 | 14.97 | 15.1 | 0.04 | 14.25 | 967 |

**Figure:** Observed versus projected regression lines of orofacial cleft incidence. Projections calculated using the Proportional Attributable Fraction for changes in active smoking prevalence in Exposure Proxy 1 (UK females) and Exposure Proxy 2 (pregnant females attending antenatal booking appointment in Scotland)**.**
