# Supplementary material for: The impact of changing cigarette smoking habits and smoke-free legislation on orofacial cleft incidence in the United Kingdom: Evidence from two time-series studies
Source: PLoS One. 2021 Nov 24;16(11):e0259820. doi: 10.1371/journal.pone.0259820 (PMC8612573; doi:10.1371/journal.pone.0259820)
Supplement: S4 Appendix — (DOCX) [file pone.0259820.s004.docx]

**S4 Appendix: Natural experiment sensitivity analyses**

**Table A:** Sensitivity analyses in England Wales and Northern Ireland for evidence of change in level of incidence in orofacial cleft (OFC), cleft lip +- palate (CLP and cleft palate only (CPO) after smoke-free legislation. The implementation of smoke-free legislation was modelled in each year where there were at least 3 data points before and after the intervention to assess chance findings for change in level. Data from the calendar year immediately following the modelled year of the ban was excluded due to involvement in the lag phase.

| **Year of ban modelled** | **OFC**  **RR (95% CI)** | **P Value** | **CLP**  **RR (95% CI)** | **P Value** | **CP**  **RR (95% CI)** | **P Value** |
| --- | --- | --- | --- | --- | --- | --- |
| 2002 | 1.04  (0.97, 1.12) | 0.275 | 1.13  (1.04, 1.23) | 0.005 | 1.13  (1.03, 1.25) | 0.014 |
| 2003 | 1.07  (1.00, 1.15) | 0.044 | 1.17  (1.08, 1.27) | 0.002 | 1.16  (1.08, 1.27) | 0.001 |
| 2004 | 1.06  (0.99, 1.4) | 0.081 | 1.15  (1.06, 1.26) | 0.002 | 1.14  (1.06, 1.23) | 0.001 |
| 2005 | 1.02  (0.95, 1.11) | 0.545 | 1.13  (1.02, 1.24) | 0.017 | 1.08  (0.97, 1.21) | 0.158 |
| 2006 | 0.98  (0.91, 1.06) | 0.588 | 1.04  (0.94, 1.15) | 0.461 | 1.05  (0.93, 1.18) | 0.461 |
| **2007*** | **0.92**  **(0.85, 0.99)** | **0.024** | **0.94**  **(0.84, 1.06)** | **0.325** | **0.97**  **(0.86, 1.08)** | **0.562** |
| 2008** | NA | NA | NA | NA | NA | NA |
| 2009 | 0.98  (0.91, 1.06) | 0.616 | 0.95  (0.85, 1.08) | 0.439 | 0.94  (0.84, 1.07) | 0.356 |
| 2010 | 1.02  (0.95, 1.11) | 0.561 | 0.97  (0.86, 1.09) | 0.586 | 0.97  (0.86, 1.09) | 0.593 |
| 2011 | 1.03  (0.95, 1.11) | 0.488 | 0.97  (0.87, 1.09) | 0.587 | 0.95  (0.85, 1.07) | 0.426 |
| 2012 | 0.99  (0.93, 1.07) | 0.921 | 0.93  (0.85, 1.02) | 0.103 | 0.91  (0.83, 1.00) | 0.057 |
| 2013 | 0.98  (0.92, 1.06) | 0.599 | 0.90  (0.82, 0.99) | 0.023 | 0.89  (0.81, 0.97) | 0.012 |
| 2014 | 0.98  (0.91, 1.06) | 0.614 | 0.90  (0.82, 0.99) | 0.037 | 0.89  (0.81, 0.98) | 0.023 |

*2007 was the actual year of ban implementation.

**2008 was not included in the sensitivity analyses as it would have analysed a level change from data excluded in the actual lag phase.

**Table B:** Sensitivity analyses in Scotland for evidence of change in level of incidence in orofacial cleft (OFC), cleft lip +- palate (CLP and cleft palate only (CPO) after smoke-free legislation. The implementation of smoke-free legislation was modelled in each year where there were at least 3 data points before and after the intervention to assess chance findings for change in level. Data from modelled year of the ban was excluded due to involvement in the lag phase.

| **Year of ban modelled** | **OFC**  **RR (95% CI)** | **P Value** | **CLP**  **RR (95% CI)** | **P Value** | **CP**  **RR (95% CI)** | **P Value** |
| --- | --- | --- | --- | --- | --- | --- |
| 2003 | 1.24  (1.11, 1.39) | 0.001 | 1.11  (0.92, 1.34) | 0.267 | 1.39  (1.42, 1.70) | 0.001 |
| 2004 | 1.27  (1.13, 1.43) | 0.001 | 1.17  (0.97, 1.41) | 0.111 | 1.40  (1.15, 1.70) | 0.001 |
| 2005 | 1.13  (0.95, 1.33) | 0.161 | 1.04  (0.85, 1.27) | 0.740 | 1.23  (0.95, 1.59) | 0.110 |
| **2006*** | **1.13**  **(0.96, 1.33)** | **0.135** | **1.01**  **(0.82, 1.25)** | **0.935** | **1.28**  **(1.01, 1.63)** | **0.045** |
| 2007** | NA | NA | NA | NA | NA | NA |
| 2008 | 0.94  (0.78, 1.2) | 0.489 | 0.93  (0.75, 1.16) | 0.505 | 0.95  (0.71, 1.28) | 0.732 |
| 2009 | 0.87  (0.72, 1.04) | 0.113 | 0.92  (0.74, 1.15) | 0.459 | 0.81  (0.61, 1.07) | 0.141 |
| 2010 | 0.83  (0.70, 0.97) | 0.020 | 1.01  (0.81, 1.25) | 0.963 | 0.68  (0.55, 0.81) | 0.001 |
| 2011 | 0.81  (0.70, 0.95) | 0.008 | 1.07  (0.86, 1.32) | 0.560 | 0.61  (0.52, 0.71) | 0.001 |
| 2012 | 0.85  (0.73, 0.99) | 0.039 | 1.08  (0.88, 1.33) | 0.436 | 0.66  (0.55, 0.78) | 0.001 |
| 2013 | 0.87  (0.74, 1.02) | 0.082 | 1.05  (0.86, 1.27) | 0.644 | 0.670.71  (0.58, 0.88) | 0.002 |
| 2014 | 0.90  (0.77, 1.05) | 0.180 | 0.99  (0.81, 1.21) | 0.856 | 0.81  (0.65, 1.01) | 0.057 |
| 2015 | 0.87  (0.74, 1.01) | 0.071 | 0.96  (o.79, 1.17) | 0.712 | 0.77  (0.61, 0.97) | 0.027 |

*****2006 was the actual year smoke-free legislation implementation.

**2007 was not included in the sensitivity analyses as it would have analysed a level change from data excluded in the actual lag phase.
