# Supplementary material for: The impact of changing cigarette smoking habits and smoke-free legislation on orofacial cleft incidence in the United Kingdom: Evidence from two time-series studies
Source: PLoS One. 2021 Nov 24;16(11):e0259820. doi: 10.1371/journal.pone.0259820 (PMC8612573; doi:10.1371/journal.pone.0259820)
Supplement: S5 Appendix — (DOCX) [file pone.0259820.s005.docx]

**S5 Appendix: Threats to external validity in the Natural Experiment**

**Table:** Time varying confounding factors that were threats to external validity for the natural experiment assessing the impact of smoke-free legislation on orofacial cleft incidence in the United Kingdom**.**

| **Time Varying Confounding Factors** | **Estimated direction of bias** | **UK Population trends** | **UK Population level events between 2000-2018** |
| --- | --- | --- | --- |
| **Smoking (other than the smoke-free legislation)** | Smoking associated with OFC | Decreasing prevalence of active smoking | 2003 UK legislation banning tobaccos advertising on billboards and printed publications  2007 – legal minimum age of tobacco increased from 16-18years in England  2009 – cigarette taxes raised 2% above inflation (previously had been in line with inflation)  2010 – Healthy Lives, Healthy People. UK Government tobacco control strategy  2017 – Towards a smoke-free generation: a tobacco control pan. UK government 5-year plan aiming to reduce prevalence of active smoking |
| **Maternal age** | Increased maternal age associated with OFC | Increasing maternal age | No national policies identified |
| **Alcohol** | Increased maternal alcohol intake associated with OFC | Trend uncertain at a population level due to challenge of measurement to encompass trends in overall alcohol consumption and binge drinking  No routine data collected on alcohol use during pregnancy at a UK level | 2003 Licensing Act in England – regulated sale and supply of alcohol  2004 Alcohol Harm Reduction Strategy for England – focus on binge drinking and chronic drinking  2007 Safe. Sensible. Social. The next steps in the national alcohol strategy in 2007 – advised pregnant women to avoid alcohol or to not exceed 1-2 units per week  2008 NICE Guidelines CG62 – pregnant women should be advised to avoid alcohol in the first trimester  2010 Alcohol Act in Scotland -regulated the sale of alcohol and licensing of premises. Chief medical officer in Scotland advised abstinence during pregnancy  2012 The Governments’ Alcohol Strategy – focus on binge drinking and alcohol-related violence.  2016 UK Chief medical officer published guidelines on alcohol consumption to encourage low risk behaviour. Pregnancy drinking advice revised to clear abstinence message for the first time |
| **Obesity** | Increased maternal body mass index associated with OFC | Increasing maternal body mass index | No national policies identified |
| **Folic acid** | Maternal folic acid supplementation associated with decreased risk of OFC | Trend uncertain due to lack of publicly available population data | No government policies identified in study period. The UK Government Department of Health made recommendations to for pregnant women to take folic acid supplementation in 1992.  The Scientific Advisory Committee on Nutrition published updated guidance on folic acid supplementation in 2006, 2009 and 2017. |
| **Ethnicity** | Changes in ethnic proportions in UK could be associated with either increased or decreased OFC risk | Trend uncertain.  Comparison between population distribution estimates in 2016 and the national census in 2011 the ethnic variation in the UK has stayed largely stable (ONS data) | No national policies identified |
| **Deprivation** | Reduced socioeconomic status of mother associated with OFC | National trend uncertain.  ONS published life expectancies by national deprivation deciles and show disparity between different areas | Global financial crash of 2008 |
