# Supplementary material for: The impact of changing cigarette smoking habits and smoke-free legislation on orofacial cleft incidence in the United Kingdom: Evidence from two time-series studies
Source: PLoS One. 2021 Nov 24;16(11):e0259820. doi: 10.1371/journal.pone.0259820 (PMC8612573; doi:10.1371/journal.pone.0259820)
Supplement: S6 Appendix — (DOCX) [file pone.0259820.s006.docx]

**S6 Appendix: Association between the incidence of orofacial cleft subtypes and active smoking prevalence**

**Table:** Ecological regression analysis to assess the correlation between the incidence of orofacial cleft subtypes cleft lip +- palate (CLP) and cleft palate only (CPO) and the proportion of active smoking in Exposure Proxy 1 (females over 16-years of age in the UK) and Exposure Proxy 2 (pregnant women in Scotland attending antenatal booking appointment). A quasi-Poisson distribution was used with adjustment for long-term trends, maternal age and a one-year lag of exposure effect.

| **Population of children born with Orofacial Cleft** | **Cleft Subtype** | **Proxy Smoking Exposure** | **Crude RR** | **P Value** | **Adjusted RR** | **P Value** |
| --- | --- | --- | --- | --- | --- | --- |
|  |  |  |  |  |  |  |
| England, Wales and Northern Ireland | CLP | 1 | 0.992  (0.984, 1.001) | 0.071 | 0.977  (0.932, 1.025) | 0.340 |
|  |  | 2 | 0.995  (0.989, 1.002) | 0.180 | 1.032  (0.959, 1.111) | 0.404 |
|  | CPO | 1 | 1.005  (0.996, 1.014) | 0.298 | 0.962  (0.926, 1.000) | 0.049 |
|  |  |  |  |  |  |  |
|  |  | 2 | 1.004  (0.997, 1.011) | 0.216 | 1.060  (1.000, 1.120) | 0.049 |
| Scotland | CLP | 1 | 0.994  (0.979, 1.009) | 0.426 | 0.963  (0.888, 1.044) | 0.362 |
|  |  | 2 | 0.998  (0.986, 1.010) | 0.715 | 1.023  (0.877, 1.194) | 0.770 |
|  | CPO | 1 | 1.014  (0.993, 1.036) | 0.189 | 1.022  (0.942, 1.108) | 0.600 |
|  |  | 2 | 1.011  (0.994, 1.028) | 0.196 | 0.963  (0.829, 1.119) | 0.624 |
