# Supplementary material for: The impact of changing cigarette smoking habits and smoke-free legislation on orofacial cleft incidence in the United Kingdom: Evidence from two time-series studies
Source: PLoS One. 2021 Nov 24;16(11):e0259820. doi: 10.1371/journal.pone.0259820 (PMC8612573; doi:10.1371/journal.pone.0259820)
Supplement: Attachment — Submitted filename: Response to Reviewers.docx [file pone.0259820.s007.docx]

Sinan Kardes, M.D.

Academic Editor

PLOS ONE

3^rd^ September 2021

Dear Sinan Kardes,

**Re:** PONE-D-21-23595

Thank you for considering our research article submission entitled **‘The impact of changing cigarette smoking habits and smoke-free legislation on orofacial cleft incidence in the United Kingdom: evidence from two time-series studies’** for publication in PLOS ONE.

Our response to each point raised by the reviewer is detailed below.

I look forward to hearing from you.

Kind regards,

**Mr Matthew Fell**

Honorary Research Fellow at the Cleft Collective, University of Bristol

Many thanks to Reviewer 1 for their kind comments and helpful suggestions.

**Major comments**

**1) Section: Methods, Statistical methods.**

**1.1.** A brief explanation of the use of the p-value should be provided. In particular, has a significance threshold been adopted? If so, specify which one. Or (recommended, <https://www.ncbi.nlm.nih.gov/pmc/articles/PMC4877414/>) were p-values used as graded measures of the strength of the evidence against the null hypothesis?

**RESPONSE**: Thank you for raising this point and for the helpful reference to Greenland et al., 2016. We resisted the use of the term ‘statistical significance’ and arbitrary dichotomous significance thresholds in the manuscript (in agreement with Greenland et al 2016 and Stern and Davey Smith 2001 https://www.ncbi.nlm.nih.gov/pmc/articles/PMC1119478). We have added an explanation of this in the statistical methods section (Page 9, lines 209-212) and hope this adds clarity.

“Effect estimates were reported as incidence rate ratios (RR) with 95% confidence intervals (CI) and p-values. P values were interpreted as continuous measures of the strength of evidence against the null hypothesis.”

**1.2.** Similarly, a brief explanation should be provided as to the strength of the correlations (e.g., RR). In particular, were “intensity” thresholds used or, in this case too, were the results evaluated using a continuous scale?

**RESPONSE**: Thank you too for this extension to the previous point. We did not use any intensity thresholds for the incidence rate ratios for the same reason of not wanting to arbitrarily dichotomise the results. Results were evaluated using a continuous scale. We hope that the added text (as per the point above) in the statistical methods (Page 9, lines 209-212) section will sufficiently address this point.

**1.3.** In some cases, in the “Results” section, the authors showed IQRs and, in others, the mean values. Was this done in consideration of the distributive nature of the data (i.e., non-Gaussians and Gaussians, respectively)? I suggest specifying this detail in the “Methods” section.

**RESPONSE**: Thank you for highlighting this confusing mix of reporting. To improve consistency, we have discarded the means and have instead reported all descriptive statistics with an interquartile range. This has been specified in the statistical methods section (page 9, lines 202-203).
“All variables were described initially through visualisation and summary statistics with interquartile range (IQR).”

**2) Section: Conclusions.** For completeness and transparency, I suggest also reporting the negative result obtained for Scotland (i.e., the absence of evident correlations).

**RESPONSE**: We have added a comment to this effect in the conclusion (page 22, lines 510-511).
“but this was not replicated in data from Scotland”.

=============

**Minor comments**

**m1) Section:** Abstract, Lines 42-46. Please also provide values for negative results.

**RESPONSE**: this has been added (page 3, line 50)

“(RR 1.16, 95%CI 0.94 to 1.44; P=0.173)”.

**m2) Section:** Methods, Lines 184-187. It is not clear why - although a decreasing trend was already present - we should not have expected a change in slope after the smoking ban implementation. In particular, why is this not supposed to have increased the reduction “speed”?

**RESPONSE**: We did not anticipate a change in slope (or ‘speed) because the smoking ban represents a singular event leading to a one-off change in passive smoking exposure. We know from data reported in this paper (i.e. active smoking prevalence and maternal age) that other factors were changing consistently during the study period, in comparison with the sudden change in passive smoking exposure following the ban. Therefore, we anticipated a change in level after the ban but not a change in slope. The anticipated model is of course, a hypothesis, but the current literature on interrupted time series specifies this impact model as an important step that should be reported. We hope that the current succinct description of this in the text is adequate.

**m3) Section:** Figures 1, 4. I suggest specifying the quantity reported on the y-axis (e.g., Figure 1, "OFC incidence [...]"). In this way, the graphs will be clear regardless of the caption.

**RESPONSE**: The y-axis on the graphs have been amended as per suggestion (Figure 1, 2 and 4).

**m4) Section:** Results, Line 276. Is a "mean of 0.8%" meant?

**RESPONSE**: Thank you. The means have now been discarded as per major comment and response above

=============

**Other comments**

**o1) Section: Methods,** Lines 130-132. I kindly ask the authors if they can better explain what they mean by this sentence. Thank you.

**RESPONSE**: The sentence has been amended. Addition of ‘due to the change in annual reporting intervals’ to improve clarity (page 6, lines 131-132).

“Children born in Scotland between January-April 2010 were counted twice due to the change in annual reporting intervals, but exclusion of 2010 data in the analysis did not impact the results.”

**o2) Section:** Methods, Lines 224-226. I kindly ask why the chosen lag was exactly one year (that is, why three months more than the gestation duration? Is this simply an upward approximation since the model works with annual rates?) Thank you.

**RESPONSE**: Yes this is correct and has been explained in the text to improve clarity (page 10, lines 230-231)

“The final model was adjusted for the yearly long-term incidence trend and

maternal age with a 1-year lag, as an upward approximation to account for the 9-month human gestation period since the model works with annual rates.”
